# Habitual coffee consumption and risk of incident heart failure: an updated systematic review and dose-response meta-analysis of prospective cohort studies

**DOI:** 10.1186/s41043-026-01295-w

**Published:** 2026-03-17

**Authors:** Shankar Biswas, Yashasvi Srivastava, Raja Kollu, Ayman Hamadttu

**Affiliations:** 1 Department of Internal Medicine, Ivano-Frankivsk National Medical University, Ivano-Frankivsk, Ukraine; 2 Department of Radiology, NMC Specialty Hospital, Abu Dhabi, United Arab Emirates; 3 Sudan University of Science and Technology, Khartoum, Sudan

**Keywords:** Coffee, Heart failure, Meta-analysis, Dose-response, Cardiovascular disease, Prospective cohort, Decaffeinated coffee

## Abstract

**Background:**

Coffee is among the most widely consumed beverages globally, yet evidence specifically examining its association with heart failure (HF) risk remains limited. The only dedicated meta-analysis on this topic was published in 2012. We conducted an updated systematic review and dose-response meta-analysis to comprehensively evaluate the association between coffee consumption and incident HF.

**Methods:**

We searched PubMed, Embase and Scopus from January 2012 through October 2025 to update the original meta-analysis. Prospective cohort studies reporting hazard ratios for coffee consumption and incident HF were included. Random-effects models were used to pool estimates. Subgroup analyses were planned to examine effects by sex, geographic region, coffee type, and population characteristics where possible. Certainty of evidence was assessed using GRADE.

**Results:**

Thirteen studies comprising 656,666 participants and 20,646 HF events were identified. Pooled analysis of 7 independent cohorts demonstrated that moderate coffee consumption (2–4 cups/day) was associated with significantly reduced HF risk (HR 0.925; 95% CI 0.882–0.971; *P* = 0.002) with negligible heterogeneity (I²=0%). A J-shaped dose-response pattern was suggested, although the test for non-linearity was borderline significant (*P* = 0.066), with maximal benefit observed at 1–2 cups/day. Within-cohort analyses indicated similar associations for caffeinated and decaffeinated coffee. No evidence of publication bias was detected (Egger’s *P* = 0.99). Certainty of evidence was rated as low.

**Conclusions:**

This updated meta-analysis suggests moderate coffee consumption is linked to lower incident heart failure risk, though certainty is low. Associations observed within-cohort analyses were similar for caffeinated and decaffeinated coffee, indicating moderate intake may be compatible within heart-healthy dietary patterns.

**Review registration:**

(PROSPERO Registration ID: CRD420251269118)

**Graphical abstract:**

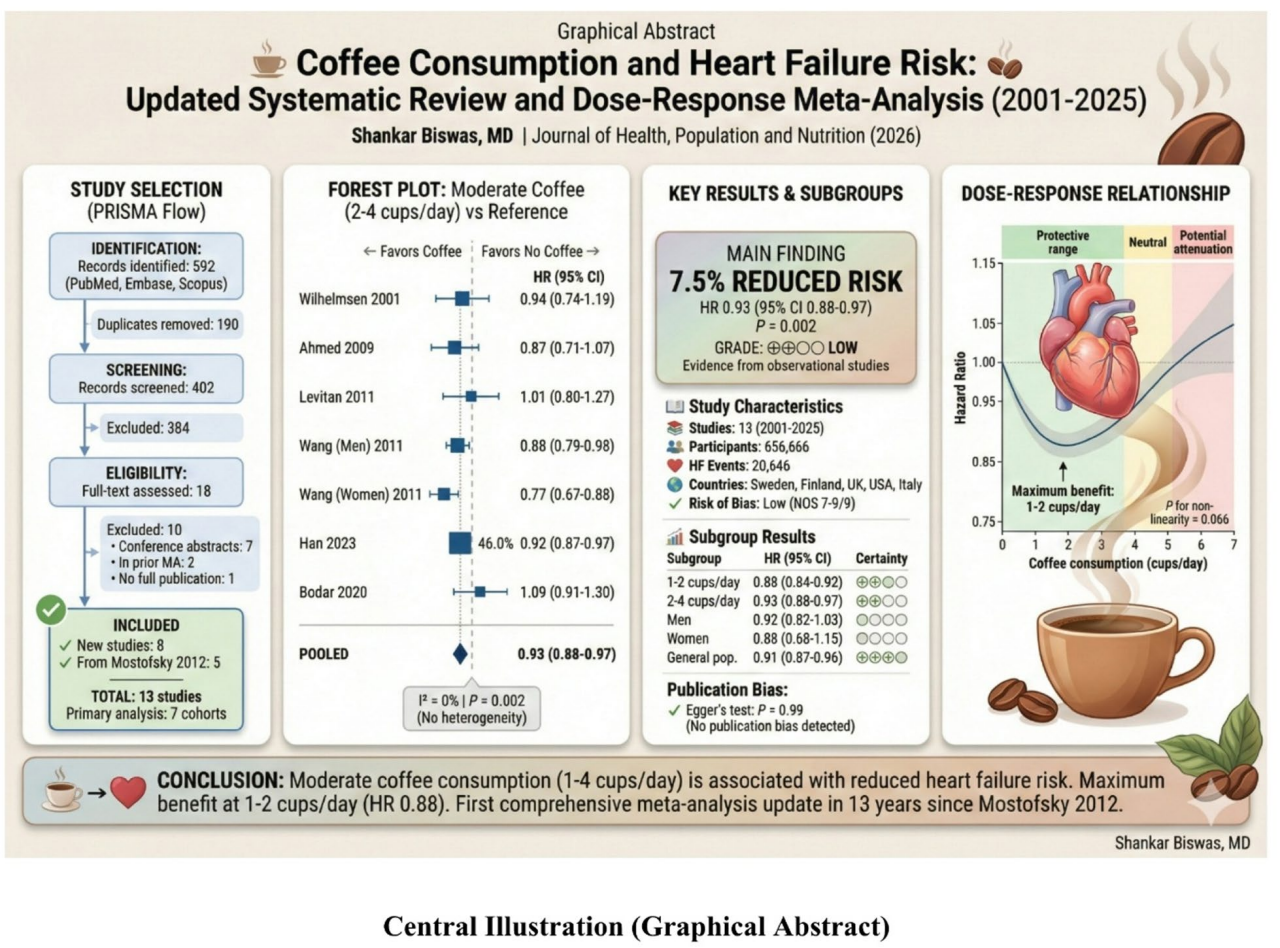

**Supplementary Information:**

The online version contains supplementary material available at 10.1186/s41043-026-01295-w.

## Background

Heart failure represents a major global public health challenge, affecting more than 64 million people worldwide and imposing substantial morbidity, mortality, and economic burden on healthcare systems [1, 2]. In the United States alone, approximately 6.7 million adults are living with heart failure, with prevalence projected to increase by 46% from 2012 to 2030 [1]. The economic costs are equally staggering, with current US expenditures exceeding $30 billion annually and projected to reach $69.8 billion by 2030 [1]. Notably, heart failure prevalence demonstrates a steep age-related gradient, rising from approximately 1% in adults younger than 55 years to more than 10% in those aged 70 years and older [3]. Given the aging global population and improved survival from acute cardiovascular events, heart failure incidence may be declining but prevalence continues to rise, creating a paradoxical expansion of the disease burden [2]. These epidemiological trends underscore the critical importance of identifying modifiable lifestyle factors that may reduce heart failure risk at the population level.

Coffee is one of the most widely consumed beverages globally, with more than 2 billion cups consumed daily [4]. Beyond caffeine, coffee contains a complex mixture of over 1,000 bioactive compounds with potential cardiovascular effects [5]. These include chlorogenic acids (50–445 mg per serving), which provide antioxidant effects and modulate nitric oxide production; diterpenes such as cafestol and kahweol (0–7 mg per serving), which regulate lipid metabolism; and trigonelline (38–540 mg per serving), which contributes to glucose homeostasis [4]. The diverse bioactive profile of coffee has generated considerable scientific interest in its potential health effects, particularly regarding cardiovascular outcomes.

Accumulating evidence supports an inverse association between moderate coffee consumption and cardiovascular disease risk. A comprehensive umbrella review found that consumption of 3–4 cups per day was associated with 17% lower all-cause mortality and 15% lower cardiovascular disease incidence [5]. However, evidence specifically examining heart failure as an outcome remains limited. A dose-response meta-analysis of 36 prospective studies encompassing over 1.2 million participants demonstrated a non-linear relationship, with the lowest cardiovascular disease risk observed at approximately 3.5 cups per day [6]. Similar protective associations have been reported for cardiovascular mortality, with meta-analytic evidence suggesting a 15% risk reduction at moderate consumption levels [7]. The biological mechanisms underlying these associations are thought to involve antioxidant and anti-inflammatory effects of chlorogenic acids, which may provide greater daily antioxidant intake than tea, fruits, and vegetables combined, as well as improvements in endothelial function and glucose metabolism [5].

Despite the extensive literature on coffee and cardiovascular disease broadly, evidence specifically examining heart failure as an outcome remains limited. The only dedicated systematic review and dose-response meta-analysis on this topic was published by Mostofsky and colleagues in 2012 [8]. That analysis included 5 prospective cohort studies comprising 140,220 participants and 6,522 heart failure events, all derived from Nordic populations (Sweden and Finland). The authors identified a statistically significant J-shaped relationship, with the strongest inverse association observed at 4 cups per day (approximately 11% lower risk) and attenuation of the protective effect at very high consumption levels [8]. However, this meta-analysis was limited by its exclusive reliance on Nordic cohorts, restricting generalizability to other populations, and by the absence of data on coffee subtypes such as ground, instant, and decaffeinated preparations.

The only dedicated systematic review and dose-response meta-analysis examining coffee consumption and heart failure risk was published by Mostofsky and colleagues in 2012 [8]. That analysis included 5 prospective cohort studies comprising 140,220 participants and 6,522 heart failure events, all derived exclusively from Nordic populations (Sweden and Finland). The authors identified a statistically significant J-shaped relationship, with the strongest inverse association observed at 4 cups per day (approximately 11% lower risk). However, this meta-analysis was limited by its exclusive reliance on Nordic cohorts, restricting generalizability, and by the absence of data on coffee subtypes.

Importantly, no research group has formally updated this meta-analysis in the intervening 13 years, despite the emergence of substantial new evidence. Large-scale prospective studies from the UK Biobank have since examined coffee consumption in relation to heart failure risk in cohorts exceeding 450,000 participants more than three times the total sample size of the original meta-analysis [9, 10]. These studies have expanded geographic representation beyond Nordic countries, introduced novel methodological approaches, and for the first time differentiated effects by coffee subtype. This significant evidence gap in the literature provides the rationale for the present updated systematic review.

We therefore conducted an updated systematic review and dose-response meta-analysis to comprehensively evaluate the association between coffee consumption and incident heart failure risk. Our objectives were to synthesize evidence from all available prospective-cohort studies published through December 2024, characterize the dose-response relationship using contemporary statistical methods, and conduct subgroup analyses by sex, geographic region, coffee subtype, and population characteristics to examine potential effect modification.

## Methods

### Study design and registration

This systematic review and dose-response meta-analysis was conducted following the Preferred Reporting Items for Systematic Reviews and Meta-Analyses (PRISMA) 2020 guidelines [11]. The protocol was registered with PROSPERO CRD420251269118 on December 18, 2025, after completion of the literature search but prior to data extraction/analysis. The study protocol was developed a priori and represents an update of the meta-analysis by Mostofsky et al. published in 2012 [8].

#### Protocol deviations

The registered protocol specified Meta-analysis of all identified studies. However, due to substantial overlap among UK Biobank publications identified during full-text review, we deviated from the original protocol by implementing a pre-specified hierarchy to select a single UK Biobank estimate for the primary analysis (Han et al. [10] 2023, We chose it as the UK Biobank representative because it had the largest sample size and most comprehensive adjustment set.). Alternative UK Biobank estimates were used only in designated subgroup analyses examining coffee subtypes and diabetic populations, with explicit acknowledgment of overlap. This deviation was documented prior to data analysis.

### Search strategy

We updated the original systematic search, which covered literature from 1966 through December 2011. For the current update, we searched PubMed, Embase, and the Scopus from January 2012 through October 2025 (The final search was conducted on October 15, 2025). The search strategy combined Medical Subject Headings (MeSH) and free-text terms related to coffee consumption (“coffee,” “caffeine,” “caffeinated beverages”) and heart failure (“heart failure,” “cardiac failure,” “ventricular dysfunction,” “cardiomyopathy”). We also manually searched reference lists of included studies and relevant review articles to identify additional eligible publications. No language restrictions were applied (Supplementary Table S1).

The original Mostofsky et al. (2012) meta-analysis searched MEDLINE (1966-December 2011) and EMBASE (1980-December 2011) using terms for coffee, caffeine, heart failure, and cardiac failure. Our updated search employed a comparable strategy with expanded database coverage (adding Scopus) and updated Medical Subject Headings. We verified that all five studies from the original meta-analysis were captured by our search strategy when applied retrospectively, confirming methodological consistency (Supplementary Table S1).

### Eligibility criteria

Studies were included if they met the following criteria: (1) prospective cohort design; (2) exposure assessment of coffee consumption (cups per day) or caffeine intake (mg per day); (3) incident heart failure as an outcome; (4) reported relative risks (RR), hazard ratios (HR), or odds ratios (OR) with 95% confidence intervals (CI) for at least three categories of coffee consumption, or sufficient data to calculate these estimates; and (5) adjustment for potential confounders including age and smoking status.

Studies were excluded if they were: (1) cross-sectional, case-control, or ecological in design; (2) conference abstracts without corresponding full-text publications; (3) duplicate reports from the same cohort without additional data; or (4) studies examining coffee consumption only in relation to mortality without incident heart failure ascertainment. When multiple publications reported data from overlapping cohorts, we selected the study with the largest sample size, longest follow-up duration, or most comprehensive adjustment for confounders.

### Data extraction

Two investigators independently extracted data using a standardized form. Discrepancies were resolved through discussion and consensus. For each study, we extracted: first author, publication year, cohort name, country, baseline period, follow-up duration, sample size, number of heart failure events, participant characteristics (age, sex distribution), exposure assessment method, exposure categories with dose midpoints, effect estimates with 95% CIs, and variables included in multivariable adjustment.

For dose-response analysis, we assigned the midpoint of each exposure category as the representative dose. For open-ended upper categories (e.g., “≥5 cups/day”), we assigned a value equal to 1.2 times the lower boundary. For open-ended lower categories (e.g., “<1 cup/day”), we assigned a value of half the upper boundary. When studies reported results separately by sex or subpopulation, we extracted data for each stratum as independent estimates.

### Risk of bias assessment

Methodological quality was assessed via 2 independent reviewers using the Newcastle-Ottawa Scale (NOS) for cohort studies [12], which evaluates three domains: selection of study groups (4 items), comparability of groups (2 items), and ascertainment of outcome (3 items). Studies scoring 7–9 were classified as low risk of bias, 4–6 as moderate risk, and 0–3 as high risk. Two reviewers independently assessed study quality, with disagreements resolved by consensus.

### Statistical analysis

We performed categorical meta-analyses comparing moderate coffee consumption (2–4 cups/day) and high consumption (≥ 5 cups/day) with the reference category (non-drinkers or lowest consumption category). Moderate consumption was defined as 2–4 cups/day to align with the categories used in the original meta-analysis by Mostofsky et al. [8], enabling direct comparison with prior findings. This range also reflects the most commonly reported intake level across included studies and it would be more appropriate to test whether 3–4 cups/day is probably not harmful, as suggested in clinical guidelines such as the European Society of Cardiology 2021 guidance [49]. Study-specific log-transformed hazard ratios and their standard errors were pooled using random-effects models [13] with restricted maximum likelihood (REML) estimation to accounts for between-study variance.

We modeled the dose-response relationship using restricted cubic splines with 3 knots at the 10th, 50th, and 90th percentiles of the exposure distribution to allow for potential non-linear associations. Non-linearity was formally tested using a likelihood ratio test comparing the spline model to a linear model. Statistical significance of the non-linearity test (*P* < 0.05) indicated that the non-linear model provided superior fit. However, we acknowledge that this approach is a crude approximation based on categorical exposure data and may not accurately capture non-linear relationships. As a sensitivity analysis, we also estimated a linear trend (hazard ratio per 1 cup/day increment) using weighted least squares regression. This linear approach assumes a monotonic relationship and may misrepresent the risk pattern if non-linearity is present; therefore, the spline-based non-linear model was considered the primary analysis.

Heterogeneity was assessed using Cochran’s Q statistic and quantified with the I² statistic, where values of 25%, 50%, and 75% represented low, moderate, and high heterogeneity, respectively [14]. We conducted prespecified subgroup analyses stratified by sex (men vs. women), geographic region (Nordic countries vs. United Kingdom vs. United States), coffee type (total, ground, decaffeinated, instant), and population type (general population vs. diabetic individuals). Differences between subgroups were evaluated using meta-regression and tests for interaction.

Sensitivity analyses included: Restricting to studies that only used never/non-drinkers as the reference group; leave-one-out analysis to assess the influence of individual studies; restriction to studies with low risk of bias (NOS ≥ 7); and exclusion of special populations (post-myocardial infarction (MI), diabetes). For studies using overlapping cohorts (particularly UK Biobank), we selected a single primary estimate for the main analysis and conducted sensitivity analyses using alternative estimates.

Publication bias was assessed visually using funnel plots and statistically using Egger’s regression test [15]. A two-sided p-value < 0.05 was considered statistically significant for all analyses.

### Certainty of evidence

Certainty of evidence was assessed using the GRADE (Grading of Recommendations, Assessment, Development and Evaluations) approach [16]. Evidence from observational studies began at low certainty and was rated down for serious limitations in risk of bias, inconsistency (I² >50%), indirectness, imprecision (confidence intervals crossing the null or fewer than 3 studies), or publication bias. Evidence was rated up for large magnitude of effect (HR < 0.5 or > 2.0), presence of a dose-response gradient, or if plausible residual confounding would reduce the observed effect. Final certainty ratings were categorized as high, moderate, low, or very low.

### Software

All statistical analyses were performed using R version 4.3.0 (R Foundation for Statistical Computing, Vienna, Austria) with the metafor package [17] for meta-analysis and the dosresmeta package for dose-response modeling.

## Results

### Study selection

**Fig. 1:**
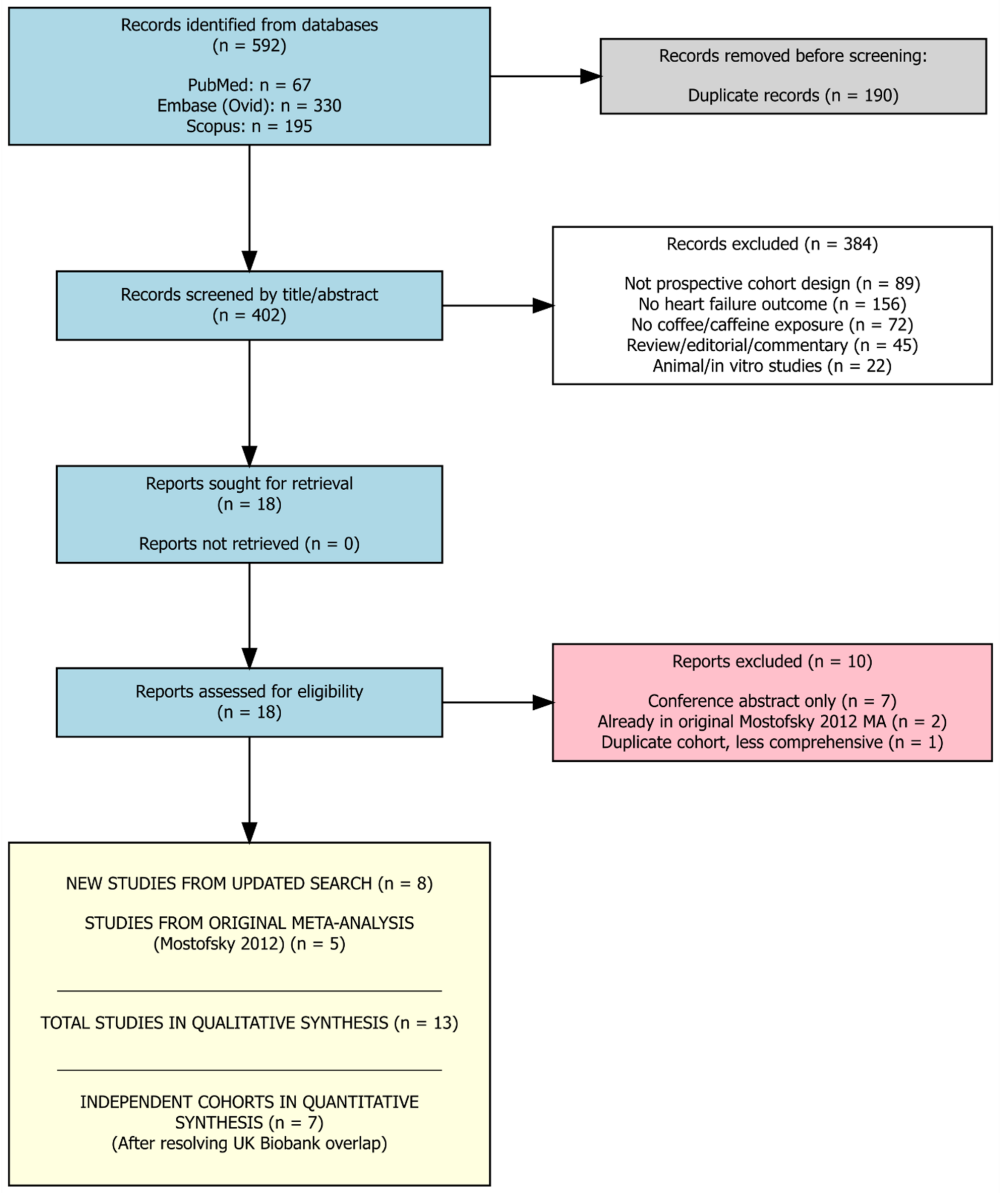
PRISMA 2020 Flow Diagram for Study Selection: Systematic review flow diagram showing identification of 592 records from three databases, with 190 duplicates removed, yielding 402 for screening. After excluding 384 records and 10 full-text articles, 8 new studies plus 5 from original Mostofsky 2012 meta-analysis were included (*n* = 13 total), with 7 independent cohorts in quantitative synthesis after resolving UK Biobank overlap

The updated literature search identified 8 new eligible studies published between 2012 and 2025 [9, 10, 23–28]. Combined with the 5 studies from the original meta-analysis by Mostofsky et al. [8], a total of 13 unique publications were included in the qualitative synthesis [9, 10, 18–28]. Seven conference abstracts were excluded as they duplicated data from full-text publications. After accounting for overlapping cohorts from the UK Biobank, 7 independent cohort analyses were included in the primary quantitative synthesis (Fig. 1; Supplementary Table S8).

Excluded studies at full-text review and included studies not used in the primary analysis are presented in Supplementary Tables S2 and S3, respectively.

### Study characteristics

The 7 independent cohorts included in the primary analysis comprised 656,666 participants and 20,646 incident heart failure events (Table 1). Studies were conducted in Sweden (*n* = 3) [18, 19, 21], Finland (*n* = 1) [22], the United Kingdom (*n* = 1) [10], and the United States (*n* = 1) [26]. Follow-up duration ranged from 8 to 35 years. Five studies enrolled participants from the general population, while two included sex-specific cohorts. For the studies included in the primary outcome, coffee consumption was assessed via validated food frequency questionnaires (FFQ) in 3 cohorts [19, 21, 26], and standardized questionnaires in 4 unique cohorts from 3 studies [10, 18, 22]. All assessments were conducted at baseline. All studies used prospective ascertainment of heart failure outcomes through medical records, hospital discharge registries, or adjudicated clinical endpoints.

**Table 1:** Characteristics of cohorts included in the primary quantitative synthesis (*n* = 7)

Study	Cohort	Country	Population / Sex	*N*	HF events	Follow-up (years)	Exposure assessment	Exposure metric	Coffee type	Categories (*n*)
Wilhelmsen [18]	Multifactor Primary Prevention Study	Sweden	General population; Men	7,374	921	26.0	Questionnaire	Cups/day	Total	3
Ahmed [19]	Cohort of Swedish Men	Sweden	No history of DM or MI; Men	37,315	784	8.0	FFQ	Cups/day	Total	5
Levitan [21]	Swedish Mammography Cohort	Sweden	No history of DM or MI; Women	34,551	602	9.0	FFQ	Cups/day	Total	5
Wang [22]	Finnish Cross-sectional Surveys (Men)	Finland	General population; Men	28,837	2,020	35.0	Questionnaire	Cups/day	Total	6
Wang [22]	Finnish Cross-sectional Surveys (Women)	Finland	General population; Women	30,653	1,807	35.0	Questionnaire	Cups/day	Total	6
Bodar [26]	Physicians Health Study	United States	Healthcare workers (physicians); Men	20,433	901	9.3	FFQ	Cups/day	Total	4
Han [10]	UK Biobank	United Kingdom	General population; Both	497,503	13,611	11.9	Questionnaire (touchscreen)	Cups/day	Total	5

*HF = heart failure.* Study-level characteristics of seven prospective cohorts included in primary quantitative synthesis, spanning Sweden, Finland, United States, and United Kingdom (2001–2023). Total of 656,666 participants with 20,646 incident heart failure events; follow-up duration ranged 8.0–35.0 years; coffee exposure assessed via questionnaire or food frequency questionnaire (FFQ)

The original Nordic studies contributed data from Swedish and Finnish populations with extended follow-up periods. Wilhelmsen et al. [18] examined 7,374 Swedish men followed for 26 years, while Ahmed et al. [19] studied 37,315 men from the Cohort of Swedish Men over 8 years. Levitan et al. [21] provided data from 34,551 women in the Swedish Mammography Cohort, and Wang et al. [22] contributed sex-stratified analyses from Finnish cross-sectional surveys encompassing nearly 60,000 participants followed for up to 35 years. The UK Biobank analysis by Han et al. [10] provided the largest single cohort (*n* = 497,503) with sophisticated time-varying exposure modeling using marginal structural models. The Physicians’ Health Study by Bodar et al. [26] contributed data from 20,433 US male physicians.

Cup sizes vary internationally, with a standard cup defined as approximately 150 mL in European studies and 240 mL (8 oz) in US studies. The UK Biobank used a standard cup of 150 mL. We extracted and analyzed data using the cup definitions reported in each original study without conversion, as dose-response relationships were established within each cohort using their respective definitions. This heterogeneity in cup size definitions is acknowledged as a limitation.

Additional studies were included in subgroup analyses. Ke et al. [23] provided sex-stratified UK Biobank data, while Liu et al. [23] and Ma et al. [27] examined diabetic populations from the UK Biobank. Tikhonoff and Casiglia [25] contributed data from an Italian cohort using dietary caffeine as the exposure. Stevens et al. [28] pooled data from the Framingham Heart Study, Atherosclerosis Risk in Communities Study (ARIC), and Cardiovascular Health Study (CHS) using machine learning approaches. Chieng et al. [9] provided the first analysis differentiating coffee subtypes (ground, instant, decaffeinated) in relation to heart failure risk.

### Risk of bias assessment

Quality assessment using the Newcastle-Ottawa Scale [12] indicated low risk of bias across included studies. Of the 7 primary cohorts, 6 scored 8–9 out of 9 points, indicating high methodological quality. One study (Wilhelmsen et al. [18]) scored 7/9 due to presentation of unadjusted effect estimates. All studies adequately defined the exposed and non-exposed cohorts, demonstrated absence of heart failure at baseline, and achieved sufficient follow-up duration. Multivariable adjustment for key confounders including age, smoking status, body mass index, and alcohol consumption was performed in all but one study (Supplementary Table S4; Supplementary Figure S1).

### Primary meta-analysis

#### Moderate coffee consumption (2–4 cups/day)

**Fig. 2:**
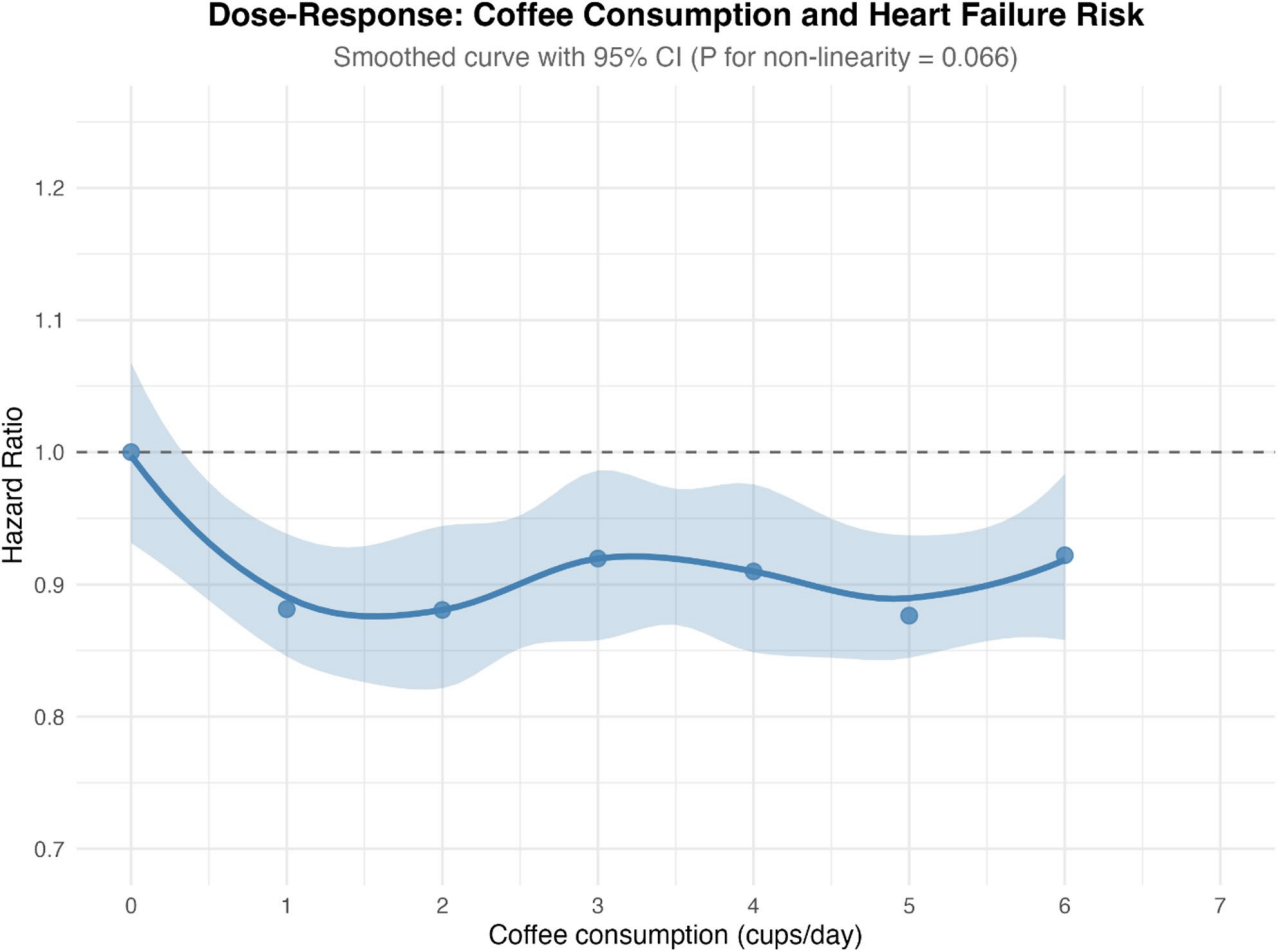
Forest Plot: Moderate Coffee Consumption (2-4 cups/day) vs. Reference: Study-specific and pooled hazard ratios for moderate coffee consumption versus non/minimal consumption (<1 cup/day). Seven cohorts contributed data; Han 2023 (UK Biobank, largest cohort) showed strongest protective effect (HR 0.92, 95% CI 0.87-0.97). Random-effects pooled estimate: HR 0.93 (95% CI 0.88-0.97), indicating 7% risk reduction with no heterogeneity (I²=0%)

Pooled analysis of 7 unique cohorts from 6 studies [10, 18, 19, 21, 22, 26] demonstrated that moderate coffee consumption (2–4 cups/day) was associated with a statistically significant 7.5% reduction in heart failure risk compared with non-drinkers or the lowest consumption category (HR 0.93; 95% CI 0.88–0.97; *P* = 0.002). Heterogeneity was negligible (I²=0.0%; Q = 6.38, *P* = 0.38), indicating consistent findings across studies (Table 2; Fig. 2).

**Table 2:** Summary of primary meta-analysis results

Analysis / Comparison	No. Cohorts	Pooled HR	95% CI	*P* value	I2
Moderate (2–4 cups/day)	7	0.925	0.882–0.971	0.0016	0.0%
High (≥ 5 cups/day)	6	0.922	0.810–1.050	0.2220	58.2%
Linear (per 1 cup/day)	5	1.010	1.002–1.018	0.0124	29.5%

*Random-effects meta-analysis.* Random-effects meta-analysis results showing protective association for moderate coffee consumption (2–4 cups/day: HR 0.925, 95% CI 0.882–0.971, *P* = 0.0016, I²=0%) and high consumption (≥ 5 cups/day: HR 0.922, 95% CI 0.810–1.050, *P* = 0.2220, I²=58.2%). Linear dose-response per 1 cup/day increase showed significant statistical association (HR 1.010, 95% CI 1.002–1.018, *P* = 0.0124, I²=29.5%) and this contradictory estimate is due to averaging across non-linear relationship

#### High coffee consumption (≥ 5 cups/day)

**Fig. 3:**
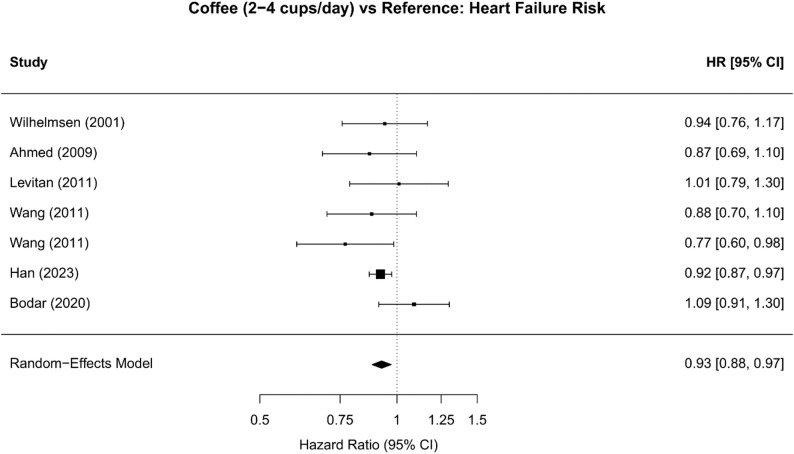
Forest Plot: High Coffee Consumption (≥5 cups/day) vs. Reference: Individual study and pooled hazard ratios for high coffee consumption showing no significant association with heart failure risk. Six cohorts included with random-effects pooled HR 0.92 (95% CI 0.81-1.05, *P*=0.222), though moderate heterogeneity present (I²=58.2%). Results suggest protective effect may not extend beyond 4 cups/day

Analysis of high coffee consumption (≥ 5 cups/day) from 6 unique cohorts from 5 studies [10, 18, 19, 21, 22] showed a similar point estimate (HR 0.92; 95% CI 0.81–1.05; *P* = 0.22), though this association did not reach statistical significance. Substantial heterogeneity was observed (I²=58.2%). This weaker and more variable association may reflect a threshold effect, consistent with the apparent J-shaped dose-response pattern, where protective benefits plateau or attenuate at higher intake levels (Table 2; Fig. 3).

#### Dose-response relationship

**Fig. 4:**
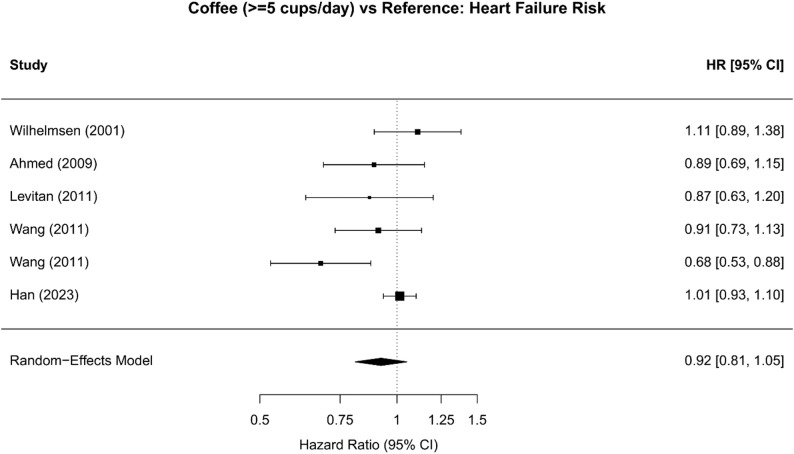
Dose–Response Relationship Between Coffee Consumption and Heart Failure Risk: Smoothed restricted cubic spline curve showing the association between daily coffee intake and heart failure risk (solid line = pooled hazard ratio; shaded area = 95% CI; reference = 0 cups/day). The curve suggests a possible J-shaped pattern, with maximal risk reduction at 1–2 cups/day (HR ≈ 0.88) and attenuation of benefit beyond 6 cups/day. However, the formal test for non-linearity was not statistically significant (*P* = 0.066). Confidence intervals widen at higher intake levels due to limited data

The dose-response curve was suggestive J-shaped non-linear relationship between coffee consumption and heart failure risk, although the formal test for non-linearity did not reach statistical significance (*P* = 0.066). Maximum risk reduction was observed at 1–2 cups per day (pooled HR approximately 0.88), with protective effects maintained through 3–4 cups per day (HR 0.91–0.92). At consumption levels exceeding 6 cups per day, the protective effect attenuated and point estimates approached the null (HR approximately 1.0). This pattern was consistent with an apparent J-shaped association and aligned with findings from Han et al. [10], who reported significantly increased risk at > 6 cups/day (HR 1.209; 95% CI 1.056–1.385) (Fig. 4; Supplementary Table S6).

### Supplementary analysis

In supplementary analysis examining consumption of 1–2 cups per day (where the dose-response nadir was observed), the pooled HR was 0.88 (95% CI 0.84–0.92; *P* < 0.0001) with no heterogeneity (I²=0%). This suggests numerically greater protection at lower-moderate consumption compared with the 2–4 cups/day range (HR 0.93), consistent with a possible J-shaped dose-response pattern, with maximum benefit at 1–2 cups per day (Table 2).

### Subgroup analyses

#### Sex-stratified analysis

The apparent protective effect of moderate coffee consumption was not statistically significant in men (4 studies contributing 5 estimates [18, 19, 22, 26]; HR 0.92, 95% CI 0.82–1.03; *P* = 0.13; I² = 35.4%). A similar pattern was observed in women (2 studies contributing 2 estimates [21, 22]; HR 0.88, 95% CI 0.68–1.15; *P* = 0.35; I² = 56.8%). The formal test for interaction by sex was also non-significant (*P* = 0.78), indicating no clear evidence of effect modification. The null finding in women may reflect limited statistical power and interpretability due to the smaller number of studies (Table 3; Supplementary Figure S3).

**Table 3:** Subgroup analyses for moderate coffee consumption (2–4 cups/day)

Subgroup	No. studies	Pooled HR	95% CI	*P* value	I2 (%)
Men	5	0.917	0.821–1.026	0.1302	35.4
Women	2	0.881	0.675–1.149	0.3507	56.8
Nordic (Sweden/Finland)	5	0.892	0.803–0.990	0.0318	0.0
General population	4	0.912	0.865–0.961	0.0005	0.0
Diabetic (T2DM)	2	N\A	N\A	N\A	N\A
Ground coffee	2	N\A	N\A	N\A	N\A
Decaffeinated coffee	2	N\A	N\A	N\A	N\A

Stratified analyses by sex, geographic region, and population type showed similar directions of effect. Moderate heterogeneity was observed in sex-stratified analyses (men I²=35.4%, women I²=56.8%), whereas no heterogeneity was detected in Nordic cohorts (I²=0%). Stratification by coffee type and diabetes status was not feasible due to overlapping cohorts; however, within-cohort analyses suggested potential benefit that requires confirmation in independent populations

In the UK Biobank (Ke et al. [23]), sex-stratified estimates at 2–4 cups/day were HR 0.93 (95% CI 0.89–0.98) for men and HR 0.98 (95% CI 0.94–1.03) for women. These within-cohort results are consistent with the absence of a meaningful sex difference but were not pooled with other studies to avoid sample overlap and unit-of-analysis error.

#### Geographic region

Stratification by geographic region revealed consistent protective associations in Nordic countries (5 studies [18–23]; HR 0.89; 95% CI 0.80–0.99; *P* = 0.032; I²=0.0%) (Table 3) and the United Kingdom (Han et al. [10]; HR 0.92; 95% CI 0.87–0.97). The single US study (Bodar et al. [26]) showed no association (HR 1.09; 95% CI 0.91–1.30), though this cohort comprised exclusively male physicians, limiting generalizability.

#### Coffee type

As coffee subtype data were available exclusively from UK Biobank publications, we present these as within-cohort findings rather than pooled meta-analytic estimates. Han et al. [10] reported HRs of 0.66 (95% CI 0.63–0.69) for ground coffee, 0.83 (95% CI 0.79–0.87) for decaffeinated coffee, and 0.89 (95% CI 0.85–0.93) for instant coffee at 1–2 cups/day compared with non-drinkers. Chieng et al. [9] reported similar patterns. These findings suggest differential effects by coffee type, with ground coffee showing the strongest association, but cannot be pooled due to cohort overlap. Although decaffeinated coffee showed similar protective associations, these results are based on a single cohort and do not permit mechanistic inference. Thus, this hypothesis requires replication in independent populations.

#### Population type

Among general population cohorts (4 studies [10, 18, 22, 26]), moderate coffee consumption was associated with a 9% risk reduction (HR 0.91; 95% CI 0.87–0.96; *P* = 0.0005; I²=0.0%) (Table 3). Two UK Biobank analyses examined diabetic populations: Liu et al. [24] reported HR 0.68 (95% CI 0.52–0.89) and Ma et al. [27] reported HR 0.89 (95% CI 0.81–0.98) for moderate consumption. As these represent overlapping participants, we do not pool these estimates but note that both suggest potential benefit in diabetic individuals, warranting investigation in independent cohorts.

### Sensitivity analyses

A linear dose-response model yielded a HR of 1.01 (95% CI 1.00-1.02; *P* = 0.012) per one cup per day increment. However, the linear model provides a simplified average estimate across the exposure range and does not capture potential non-linearity suggested by the data. When the underlying relationship is non-linear, a linear model averages across protective effects at low-to-moderate consumption and attenuated effects at higher intake. The test for non-linearity comparing spline and linear models yielded *P* = 0.066. While borderline, the visual J-shaped pattern, stronger effect at 1–2 cups/day (HR 0.88) versus 2–4 cups/day (HR 0.93), and attenuation beyond 6 cups/day are consistent with a possible J-shaped pattern, although statistical evidence for non-linearity was borderline (*P* = 0.066). The limited number of pooled dose categories may have reduced power to detect non-linearity statistically. Additionally, the positive linear coefficient reflects averaging across the exposure distribution and should be interpreted cautiously in the context of the observed non-linear pattern (Supplementary Table S7).

Leave-one-out analysis demonstrated stability of the primary findings. Sequential exclusion of individual studies yielded pooled hazard ratios ranging from 0.91 to 0.93, with all estimates remaining statistically significant. Removal of the Han et al. [10] UK Biobank study, which contributed the largest weight, widened confidence intervals (HR 0.93; 95% CI 0.84–1.04) but did not materially alter the direction of effect. The I² statistic increased to 24.2% upon exclusion of this study, suggesting it contributed to overall consistency of findings. In a sensitivity analysis restricted to four unique cohorts from three studies [18, 22, 26]-including two independent estimates from Wang et al. [22], reported separately for men and women-and using never/non-drinkers as the reference group (excluding those consuming < 1 cup/day or < 1 cup/week; Supplementary Table S9), the pooled HR for moderate consumption was 0.93 (95% CI, 0.80–1.07; *P* = 0.31; I² = 45.8%).Although not statistically significant-likely due to the smaller sample size-this result was consistent with the primary analysis and supports the robustness of the findings to variations in reference group definition.

### Publication bias

Visual inspection of the funnel plot revealed no asymmetry suggestive of publication bias. Egger’s regression test [15] confirmed the absence of small-study effects (z = 0.013; *P* = 0.99). These findings indicate that the observed protective association is unlikely to be attributable to selective publication of positive results (Supplementary Figure S2).

### Certainty of evidence

The GRADE certainty of evidence assessment is summarized in Supplementary Table S5. For the primary outcome of moderate coffee consumption (2–4 cups/day) and heart failure risk, the certainty of evidence was rated as **LOW**. Observational studies start at low certainty, and the evidence was not upgraded because the observed J-shaped pattern did not reach statistical significance for non-linearity. Furthermore, the formal test for non-linearity was borderline (*P* = 0.066), providing insufficient statistical evidence to definitively characterize the dose-response shape. We therefore did not apply an upgrade criterion. We considered downgrading for residual confounding but judged it unlikely to fully explain the consistency of results. Additionally, risk of bias was low across studies (NOS 7–9/9), heterogeneity was negligible (I² = 0%), the evidence was direct, estimates were precise, and no publication bias was detected.

For high coffee consumption (≥ 5 cups/day), the certainty of evidence was rated as **VERY LOW**, downgraded for serious inconsistency (I² = 58.2%) and imprecision (confidence interval crossing the null). For men evidence was rated as **VERY LOW** due to imprecision. Similarly, evidence for the association in women was rated as **VERY LOW** due to moderate heterogeneity (I² = 57%), imprecision, and a limited number of studies (*n* = 2).

Among subgroup analyses, **MODERATE** certainty was achieved for general population cohorts (4 studies; upgraded for consistency as I² = 0%). Evidence for ground coffee, decaffeinated coffee, and diabetic populations was not rated, as meta-analysis was not applicable.

## Discussion

### Summary of main findings

This updated systematic review and dose-response meta-analysis demonstrates that moderate coffee consumption is associated with a statistically significant reduction in heart failure risk. Pooled analysis of 7 independent prospective cohorts comprising 656,666 participants and 20,646 heart failure events revealed that consumption of 2–4 cups per day was associated with a 7.5% lower risk of incident heart failure compared with non-drinkers (HR 0.93; 95% CI 0.88–0.97; *P* = 0.002), with negligible heterogeneity (I²=0%). The dose-response relationship appeared J-shaped, with the test for non-linearity bordering on significance (*P* = 0.066). Maximum benefit was observed at 1–2 cups per day, with protective effects attenuating at consumption levels exceeding 6 cups per day. Notably, both caffeinated and decaffeinated coffee were inversely associated with heart failure risk in within-cohort analyses, suggesting that non-caffeine bioactive compounds may contribute to cardiovascular protection.

### Comparison with prior evidence

To our knowledge, this represents the first comprehensive update of the coffee-heart failure evidence base since the seminal meta-analysis by Mostofsky and colleagues in 2012 [8]. Exhaustive searches confirm that no dedicated meta-analysis on coffee and heart failure has been published in the intervening 13 years [29, 30], despite the emergence of substantial new evidence from large prospective-cohorts. Our findings are consistent with the original meta-analysis, which identified a suggestive J-shaped relationship with approximately 11% risk reduction at 4 cups per day [8]. The present analysis extends these findings by incorporating data from geographically diverse populations, nearly quintupling the sample size, and providing the first synthesis of evidence on coffee subtypes.

Our results align with the broader literature on coffee and cardiovascular health. Umbrella reviews and comprehensive assessments have consistently demonstrated inverse associations between moderate coffee consumption and cardiovascular disease incidence, cardiovascular mortality, and all-cause mortality [5, 29, 31]. The landmark New England Journal of Medicine review concluded that 3–5 cups per day is consistently associated with approximately 15% reduced cardiovascular risk [32]. The machine learning analysis by Stevens et al., which pooled data from the Framingham Heart Study, ARIC, and Cardiovascular Health Study, reported a hazard ratio of 0.95 per cup per day for heart failure, closely concordant with our pooled estimate [28].

### Biological plausibility

The cardioprotective effects of coffee are biologically plausible and supported by extensive mechanistic evidence. Coffee contains over 1,000 bioactive compounds, including chlorogenic acids (50–445 mg per serving), which exert antioxidant and anti-inflammatory effects through inhibition of NF-κB signaling and activation of the Nuclear Factor Kappa B over Heme Oxygenase-1 (Nrf2/HO-1) pathway [4, 33, 34]. These mechanisms directly target established pathways in heart failure pathophysiology, where oxidative stress and chronic inflammation drive myocardial remodeling and disease progression [35, 36].

A particularly noteworthy finding is the significant protective association observed for decaffeinated coffee (Han et al. [10] reported an HR of 0.83 (95% CI 0.79–0.87), and Chieng et al. [9] reported a similar pattern.), which may suggest that caffeine is not the primary mediator of cardiovascular benefit, even though that these findings cannot suggest a mechanistic inference. Decaffeination removes caffeine but preserves chlorogenic acids, trigonelline, polyphenols, and melanoidins compounds with demonstrated cardioprotective properties [4, 37]. Recent evidence from the largest study to date on coffee and gut microbiota demonstrated that both caffeinated and decaffeinated coffee promote growth of beneficial intestinal bacteria through caffeine-independent mechanisms [38]. These gut microbiome effects may contribute to cardiovascular protection via short-chain fatty acid production and systemic anti-inflammatory signaling.

### Dose-response relationship

The J-shaped dose-response pattern, with benefit at moderate consumption and attenuation at very high intake, has been consistently observed across cardiovascular outcomes [39] and is mechanistically explicable. At moderate doses (1–4 cups/day), the beneficial effects of polyphenols and chlorogenic acids predominate. At very high doses (> 6 cups/day), caffeine-mediated sympathetic activation, sleep disruption, and potential pro-arrhythmic effects may counterbalance these benefits [40, 41]. The European Food Safety Authority (EFSA) has established 400 mg of caffeine daily (approximately 4–5 cups) as the safe threshold for healthy adults, which aligns with the inflection point observed in our dose-response curve [40].

Although our primary categorical analysis focused on 2–4 cups/day to align with prior literature, the dose-response analysis suggests that maximum benefit may occur at 1–2 cups/day. This has practical implications: even modest coffee consumption (1–2 cups/day) may confer cardiovascular benefit, while increasing intake to 3–4 cups/day provides diminishing returns rather than additional protection.

### Interpretation of subgroup findings

The protective association was not statistically significant in both men and women. The formal test for interaction was non-significant (*P* = 0.78), suggesting this difference reflects statistical power rather than true biological effect modification. This interpretation is supported by the UK Biobank analysis of nearly 500,000 participants, which found no sex differences in coffee-mortality associations (*P* = 0.45 for heterogeneity) [42]. While Cytochrome P450 1A2 (CYP1A2) enzyme activity, which mediates caffeine metabolism, is higher in men than women [43], this pharmacokinetic difference does not appear to translate to differential cardiovascular outcomes at the population level.

The null finding in the Physicians’ Health Study (HR 1.09) may reflect several factors. First, this cohort comprised exclusively male physicians who may have lower baseline cardiovascular risk and less variability in health behaviors, resulting in attenuated exposure-outcome associations due to restriction of range (rather than healthy user bias, which would bias away from the null [44]). Second, the US population may have different coffee preparation practices compared with Nordic and UK populations, where filtered coffee predominates [45, 46]. Third, this could represent genuine population heterogeneity or chance finding. We acknowledge that the reason for this discrepant result remains uncertain and warrants investigation in other US cohorts.

The substantial heterogeneity observed within-cohort for ground coffee warrants careful interpretation. The source of heterogeneity could not be identified due to limited studies and lack of standardized reporting of brewing methods. Potential contributors may be due to methodological imprecision inherent in the “ground coffee” category, which encompasses fundamentally different brewing methods including: filtered drip, French press, espresso, and Turkish/boiled coffee that produce 3-6-fold differences in bioactive compound extraction [47]. Filtered methods remove cholesterol-raising diterpenes while preserving cardioprotective polyphenols, whereas unfiltered methods retain diterpenes that may partially offset cardiovascular benefits. Future studies should more precisely characterize brewing methods to reduce this source of heterogeneity. Given this substantial heterogeneity, the pooled estimate for ground coffee should be interpreted with caution (GRADE: very low certainty). In contrast, decaffeinated coffee demonstrated no heterogeneity (I²=0%), likely reflecting more standardized industrial processing [37].

The trend toward stronger protection in diabetic populations (Liu et al. reported HR 0.68 [95% CI 0.52–0.89] and Ma et al. reported HR 0.89 [95% CI 0.81–0.98] for moderate consumption) should be interpreted cautiously. The small number of studies and heterogeneity among estimates preclude firm conclusions, and confirmation in independent cohorts is required. These findings should therefore be considered exploratory. Although potential mechanisms related to glucose metabolism and insulin sensitivity have been proposed in prior literature [48], the present data are insufficient to support mechanistic inferences.

### Strengths and limitations

Strengths of this meta-analysis include the large pooled sample size (656,666 participants; 20,646 events), geographic expansion beyond Nordic countries, the first, to our knowledge, synthesis of coffee subtype data, low heterogeneity in the primary analysis, absence of publication bias, and consistent findings across sensitivity analyses.

Several limitations merit consideration. First, as with all observational evidence, residual confounding cannot be excluded despite multivariable adjustment in included studies. Coffee consumption correlates with lifestyle factors that may independently influence heart failure risk. Second, most studies assessed coffee consumption at a single baseline timepoint, which may not capture changes in consumption patterns over follow-up. Third, cup sizes vary internationally, introducing measurement heterogeneity. Fourth, we could not examine effects of coffee additives (sugar, cream) which may modify health effects.

An important limitation is the potential for residual confounding by overall diet quality and lifestyle patterns. Individuals who consume moderate amounts of coffee may differ systematically from non-drinkers and heavy consumers in ways that independently affect cardiovascular risk. For example, moderate coffee consumption may correlate with adherence to healthier dietary patterns (e.g., Mediterranean diet), regular physical activity, moderate alcohol intake, and higher socioeconomic status. While included studies adjusted for major confounders (age, sex, smoking, BMI, alcohol, physical activity), none fully adjusted for overall diet quality indices. It is therefore possible that observed associations reflect broader lifestyle patterns rather than causal effects of coffee per se. This confounding structure is difficult to address in observational studies and represents a fundamental limitation of the evidence base.

Reference group definitions varied across studies, with some using never-drinkers and others using lowest consumption categories (< 1 cup/day or < 1 cup/week). If very low consumption confers some protection, combining these groups may bias the reference toward higher risk, potentially inflating the apparent protective effect of moderate consumption. Sensitivity analysis restricted to studies using never-drinkers as reference yielded similar results (HR 0.93), providing some reassurance, though this analysis had reduced statistical power.

Finally, the UK Biobank contributed several overlapping analyses, which complicated subgroup analyses (such as those by coffee type and population). As a result, these subgroup findings were largely descriptive and required careful selection to avoid double-counting participants and to ensure appropriate interpretation of within-cohort results.

### Clinical implications

Our findings support the position that moderate coffee consumption (2–4 cups/day) can be incorporated into a heart-healthy lifestyle. This aligns with the European Society of Cardiology guidelines, which state that moderate coffee consumption is “probably not harmful and perhaps even moderately beneficial” for cardiovascular health [49], and the Dietary Guidelines for Americans, which include coffee as an acceptable beverage within healthy dietary patterns [50]. However, given the low certainty of evidence and potential for residual confounding, these findings should not be interpreted as definitive proof of causation. Current guidelines do not specifically address heart failure our findings contribute to this evidence gap but do not warrant changes to clinical recommendations pending higher-quality evidence.

The finding that decaffeinated coffee confers similar protection has practical implications for individuals who wish to limit caffeine intake due to sleep disturbance, anxiety, or arrhythmia concerns. Our results suggest that switching to decaffeinated coffee would preserve cardiovascular benefits while avoiding caffeine-related side effects.

### Future directions

Although randomized controlled trials of coffee consumption and heart failure outcomes are unlikely to be feasible, Mendelian randomization studies using genetic instruments for coffee consumption could strengthen causal inference [30, 42]. Subsequent research should specifically observe the 1–2 cups/day group, as they may derive the greatest cardiovascular benefit. Future observational research should more precisely characterize brewing methods and coffee subtypes to reduce heterogeneity. Biomarker studies examining the effects of coffee on inflammatory markers, oxidative stress indices, and cardiac biomarkers (natriuretic peptides, troponins) would further elucidate mechanisms. Finally, studies in diverse populations, particularly those underrepresented in current evidence (Asian, African, Latin American populations), would enhance generalizability.

## Conclusions

This updated meta-analysis suggests that moderate coffee consumption is associated with a lower risk of incident heart failure, although the certainty of evidence is low. The findings are compatible with a J-shaped dose–response pattern, but statistical evidence for non-linearity was borderline. Within-cohort analyses indicated similar associations for caffeinated and decaffeinated coffee. While emphasizing the need for confirmation in independent populations. These findings, representing the first comprehensive synthesis of evidence in 13 years, suggest that moderate coffee consumption can be part of a heart-healthy dietary pattern, while emphasizing the need for confirmation in independent populations.

## Supplementary Information

Supplementary Material 1.

## Data Availability

All data extracted for this systematic review are available in the manuscript and supplementary materials. The study protocol is registered and publicly available at PROSPERO (https:/www.crd.york.ac.uk/PROSPERO/view/CRD420251269118). Statistical code is available from the corresponding author upon reasonable request. No individual patient data were accessed for this analysis.

## Abbreviations

ARIC: Atherosclerosis Risk in Communities Study
CHS: Cardiovascular Health Study
CYP1A2: Cytochrome P450 1A2 (enzyme)
EFSA: European Food Safety Authority
GRADE: Grading of Recommendations, Assessment, Development and Evaluations
HF: Heart Failure
HO-1: Heme Oxygenase-1
MI: Myocardial Infarction
NF-κB: Nuclear Factor Kappa B
NOS: Newcastle-Ottawa Scale
Nrf2: Nuclear factor erythroid 2-related factor 2
PRISMA: Preferred Reporting Items for Systematic Reviews and Meta-Analyses
PROSPERO: International Prospective Register of Systematic Reviews
REML: Restricted Maximum Likelihood
