# Supplementary material for: Habitual coffee consumption and risk of incident heart failure: an updated systematic review and dose-response meta-analysis of prospective cohort studies
Source: J Health Popul Nutr. 2026 Mar 17;45:119. doi: 10.1186/s41043-026-01295-w (PMC13112914; doi:10.1186/s41043-026-01295-w)
Supplement: Supplementary file 1 — Supplementary Material 1. [file 41043_2026_1295_MOESM1_ESM.docx]

**SUPPLEMENTARY MATERIALS**

**Table of Content:**A\ List of Supplementary Tables: (Page 1 – 12) (7 Supplementary Tables)
B\ Supplementary Figures and Figures’ Legends (Page 12 – 15) (3 Supplementary Figures)

**A\ List of Supplementary Tables**

**Supplementary Table S1.** Search Strategy by Database and comparison with the reference Meta-analysis

| Database | Search strategy | | Date searched | Results |
| --- | --- | --- | --- | --- |
| PubMed/MEDLINE | (("Coffee"[Mesh] OR "coffee"[tiab] OR "caffeine"[tiab] OR "caffeinated beverage"[tiab] OR "caffeinated beverages"[tiab] OR "espresso"[tiab] OR "cappuccino"[tiab] OR "decaffeinated coffee"[tiab] OR "instant coffee"[tiab] OR "ground coffee"[tiab] OR "filtered coffee"[tiab] OR "unfiltered coffee"[tiab])) AND (("Heart Failure"[Mesh] OR "heart failure"[tiab] OR "cardiac failure"[tiab] OR "congestive heart failure"[tiab] OR "CHF"[tiab] OR "HF"[tiab] OR "HFrEF"[tiab] OR "HFpEF"[tiab] OR "heart decompensation"[tiab] OR "ventricular dysfunction"[tiab] OR "systolic heart failure"[tiab] OR "diastolic heart failure"[tiab] OR "acute heart failure"[tiab] OR "chronic heart failure"[tiab])) AND (("Cohort Studies"[Mesh] OR "Prospective Studies"[Mesh] OR "Follow-Up Studies"[Mesh] OR "Longitudinal Studies"[Mesh] OR "cohort"[tiab] OR "prospective"[tiab] OR "longitudinal"[tiab] OR "follow-up"[tiab] OR "follow up"[tiab] OR "incidence"[tiab] OR "incident"[tiab] OR "observational"[tiab])) NOT (("Animals"[Mesh] NOT "Humans"[Mesh])) | | October 2025 | 67 |
| Embase (Ovid) | ('coffee'/exp OR 'coffee':ab,ti OR 'caffeine'/exp OR 'caffeine':ab,ti OR 'caffeinated beverage':ab,ti OR 'caffeinated beverages':ab,ti OR 'espresso':ab,ti OR 'cappuccino':ab,ti OR 'decaffeinated coffee':ab,ti OR 'instant coffee':ab,ti OR 'ground coffee':ab,ti OR 'filtered coffee':ab,ti OR 'unfiltered coffee':ab,ti) AND ('heart failure'/exp OR 'congestive heart failure'/exp OR 'heart failure':ab,ti OR 'cardiac failure':ab,ti OR 'congestive heart failure':ab,ti OR 'chf':ab,ti OR 'hf':ab,ti OR 'hfref':ab,ti OR 'hfpef':ab,ti OR 'heart decompensation':ab,ti OR 'ventricular dysfunction':ab,ti OR 'systolic heart failure':ab,ti OR 'diastolic heart failure':ab,ti OR 'acute heart failure':ab,ti OR 'chronic heart failure':ab,ti) AND ('cohort analysis'/exp OR 'prospective study'/exp OR 'follow up'/exp OR 'longitudinal study'/exp OR 'cohort':ab,ti OR 'prospective':ab,ti OR 'longitudinal':ab,ti OR 'follow-up':ab,ti OR 'follow up':ab,ti OR 'incidence':ab,ti OR 'incident':ab,ti OR 'observational':ab,ti) NOT ('animal'/exp NOT 'human'/exp) | | October 2025 | 330 |
| Scopus | TITLE-ABS-KEY(("coffee" OR "caffeine" OR "caffeinated beverage" OR "caffeinated beverages" OR "espresso" OR "cappuccino" OR "decaffeinated coffee" OR "instant coffee" OR "ground coffee" OR "filtered coffee" OR "unfiltered coffee")) AND TITLE-ABS-KEY(("heart failure" OR "cardiac failure" OR "congestive heart failure" OR "CHF" OR "HF" OR "HFrEF" OR "HFpEF" OR "heart decompensation" OR "ventricular dysfunction" OR "systolic heart failure" OR "diastolic heart failure" OR "acute heart failure" OR "chronic heart failure")) AND TITLE-ABS-KEY(("cohort" OR "prospective" OR "longitudinal" OR "follow-up" OR "follow up" OR "incidence" OR "incident" OR "observational")) AND NOT TITLE-ABS-KEY((animal* NOT human*)) | | October 2025 | 195 |
| Total records identified from databases | 592 | | | |
| Duplicate records removed before screening | 190 | | | |
| Records screened (title/abstract) | 402 | | | |
| Search Strategy Comparison | | | | |
| Element | **Mostofsky 2012** | **Current Update** | | |
| Databases | MEDLINE, EMBASE | PubMed, Embase, Scopus, Cochrane | | |
| Search period | 1966-Dec 2011 | Jan 2012-Oct 2025 | | |
| Search date | Not reported | October 15, 2025 | | |
| Search terms | Coffee, caffeine, heart failure, cardiac failure | Coffee, caffeine, caffeinated beverages, heart failure, cardiac failure, ventricular dysfunction, cardiomyopathy | | |
| Language restrictions | None | None | | |

Detailed search strategies for PubMed/MEDLINE, Embase (Ovid), and Scopus including all subject headings, keywords, and Boolean operators used. Search conducted January 2025, yielding 592 records before deduplication (67 from PubMed, 330 from Embase, 195 from Scopus), with 190 duplicates removed. Comparison of the search strategy with the reference meta-analysis demonstrated improvements across nearly all elements.

**Supplementary Table S2.** Studies Excluded at Full-Text Review with Reasons (n = 10)

| # | First Author | Year | Title/Description | Reason for Exclusion |
| --- | --- | --- | --- | --- |
| 1 | Bodar V | 2018 | Coffee consumption and risk of heart failure in the Physicians' Health Study (ESC Congress abstract) | Conference abstract; superseded by full publication (Bodar 2020) |
| 2 | Tikhonoff V | 2017 | Dietary caffeine and cardiovascular risk: preliminary results of a longitudinal study (ESH abstract) | Conference abstract; superseded by full publication (Tikhonoff 2023) |
| 3 | Stevens L | 2018 | Machine learning analysis of coffee consumption and incident heart failure (AHA abstract) | Conference abstract; superseded by full publication (Stevens 2021) |
| 4 | Chieng D | 2021 | Coffee subtypes and cardiovascular outcomes in UK Biobank (CSANZ abstract) | Conference abstract; superseded by full publication (Chieng 2022) |
| 5 | Han Q | 2022 | Coffee consumption and heart failure: UK Biobank analysis (preliminary report) | Conference abstract; superseded by full publication (Han 2023) |
| 6 | Ke D | 2024 | Sex differences in beverage consumption and cardiovascular disease (conference presentation) | Conference abstract; superseded by full publication (Ke 2025) |
| 7 | Liu Y | 2022 | Coffee and cardiovascular outcomes in diabetic patients (preliminary data) | Conference abstract; superseded by full publication (Liu 2023) |
| 8 | Ahmed HN | 2009 | Coffee consumption and risk of heart failure in men: an analysis from the Cohort of Swedish Men | Already included in original Mostofsky 2012 meta-analysis |
| 9 | Levitan EB | 2011 | Coffee consumption and incidence of heart failure in women | Already included in original Mostofsky 2012 meta-analysis |
| 10 | Krittanawong C | 2018 | Association between coffee consumption and risk of heart failure: a systematic review and meta-analysis | Conference abstract only; no full peer-reviewed publication available |

**Summary of Exclusion Reasons**

| Reason | Number of Studies |
| --- | --- |
| Conference abstract only (excluded at full text) | 7 |
| Already in original Mostofsky 2012 meta-analysis | 2 |
| Duplicate cohort, less comprehensive (overlap) | 1 |
| Total full-text exclusions | **10** |

Complete list of 10 studies excluded at full-text review with first author, year, study description, and specific exclusion rationale. Primary exclusion categories: conference abstracts superseded by full publications (n=7), already included in original Mostofsky 2012 meta-analysis (n=2), and duplicate cohort with less comprehensive data (n=1).

**Supplementary Table S3.** Included studies not used in the primary quantitative synthesis

| Study | Cohort | Reason excluded from primary synthesis | Used in |
| --- | --- | --- | --- |
| Ke 2025 | UK Biobank (men/women) | Overlaps with Han 2023 UK Biobank (primary); sex-stratified only | Sex subgroup analysis (descriptive within-cohort only) |
| Liu 2023 | UK Biobank (T2DM subset) | Overlaps with Han 2023; special population subset | Diabetic population subgroup  (descriptive within-cohort only) |
| Ma 2025 | UK Biobank (T2DM subset) | Overlaps with Han 2023; special population subset | Diabetic population subgroup (descriptive within-cohort only) |
| Chieng 2022 | UK Biobank | Overlaps with Han 2023; subtype analysis | Coffee type subgroup (descriptive within-cohort only) |
| Stevens 2021 | FHS/ARIC/CHS pooled | Continuous exposure only (per-cup); not comparable to categorical pooling | Qualitative and sensitivity |
| Mukamal 2009 | SHEEP / post-MI cohort | Special population (post-myocardial infarction) | Sensitivity only |
| Tikhonoff 2023 | Italian cohort | Caffeine exposure (mg/day) rather than coffee cups/day | Qualitative / caffeine subgroup |

Six studies excluded from primary meta-analysis due to overlapping UK Biobank populations or special subgroups, with detailed reasons and their use in secondary analyses. Han 2023 selected as primary UK Biobank study; others used for subgroup analyses (sex-stratified, diabetic populations, coffee subtypes).

**Supplementary Table S4.** Risk of bias (Newcastle-Ottawa Scale) summary

| Study | Cohort / stratum | NOS (0-9) | Quality rating | Notes |
| --- | --- | --- | --- | --- |
| Wilhelmsen 2001 | Multifactor Primary Prevention Study | 7 | Low risk | No adjustment for confounders |
| Ahmed 2009 | Cohort of Swedish Men | 9 | Low risk | Well-adjusted model |
| Ahmed 2009 | Cohort of Swedish Men (DM/MI) | 9 | Low risk | DM/MI subgroup analysis |
| Mukamal 2009 | Stockholm Heart Epidemiology Program | 8 | Low risk | Post-MI population only |
| Levitan 2011 | Swedish Mammography Cohort | 9 | Low risk | Well-adjusted model |
| Wang 2011 | Finnish Cross-sectional Surveys (Men) | 9 | Low risk | Well-adjusted model |
| Wang 2011 | Finnish Cross-sectional Surveys (Women) | 9 | Low risk | Well-adjusted model |
| Bodar 2020 | Physicians Health Study | 8 | Low risk | Male physicians only; limited generalizability |
| Stevens 2021 | Framingham Heart Study | 9 | Low risk | FHS cohort; machine learning feature selection |
| Stevens 2021 | ARIC | 9 | Low risk | ARIC cohort; caffeine exposure |
| Stevens 2021 | Cardiovascular Health Study | 9 | Low risk | CHS cohort |
| Chieng 2022 | UK Biobank (Total) | 9 | Low risk | UK Biobank total coffee |
| Chieng 2022 | UK Biobank (Ground) | 9 | Low risk | Ground coffee subtype |
| Chieng 2022 | UK Biobank (Decaf) | 9 | Low risk | Decaffeinated coffee subtype |
| Chieng 2022 | UK Biobank (Instant) | 9 | Low risk | Instant coffee subtype |
| Han 2023 | UK Biobank | 9 | Low risk | Marginal structural models; time-varying exposure |
| Han 2023 | UK Biobank (Decaf) | 9 | Low risk | Decaffeinated coffee subtype |
| Han 2023 | UK Biobank (Ground) | 9 | Low risk | Ground coffee subtype |
| Liu 2023 | UK Biobank (T2DM never-smokers) | 8 | Low risk | Selected T2DM never-smokers; UK Biobank subset |
| Tikhonoff 2023 | Italian Population Cohort | 9 | Low risk | Dietary diary; caffeine from all sources |
| Tikhonoff 2023 | Italian Population Cohort (Men) | 9 | Low risk | Men only subgroup |
| Ke 2025 | UK Biobank (Men) | 9 | Low risk | UK Biobank men; self-reported exposure |
| Ke 2025 | UK Biobank (Women) | 9 | Low risk | UK Biobank women; self-reported exposure |
| Ma 2025 | UK Biobank (T2DM) | 8 | Low risk | UK Biobank T2DM subset |

Network structure summary showing 8 treatment nodes, 9 studies, and insufficient closed loops for formal node-splitting inconsistency assessment. Assessment timestamp: November 4, 2025, 8:02 AM; recommendation indicates network unsuitable for traditional inconsistency analysis due to star-shaped geometry.

**Supplementary Table S5.** GRADE summary of findings

| Outcome | No. studies | Participants | Effect (HR, 95% CI) | I2 | Certainty |
| --- | --- | --- | --- | --- | --- |
| Moderate coffee (2-4 cups/day) | 7 | 656,666 | 0.925 (0.882-0.971) | 0% | ⊕⊕◯◯ LOW |
| High coffee (>=5 cups/day) | 6 | ~600,000 | 0.922 (0.810-1.050) | 58% | ⊕◯◯◯ VERY LOW |
| Men - moderate coffee | 5 | - | 0.917 (0.821-1.026) | 35.4% | ⊕◯◯◯ VERY LOW |
| Women - moderate coffee | 2 | - | 0.881 (0.675-1.149) | 56.8% | ⊕◯◯◯ VERY LOW |
| General population | 4 | ~570,000 | 0.912 (0.865-0.961) | 0% | ⊕⊕⊕◯ MODERATE |

GRADE certainty: ⊕⊕⊕⊕ High; ⊕⊕⊕◯ Moderate; ⊕⊕◯◯ Low; ⊕◯◯◯ Very Low Evidence upgraded for dose-response gradient; downgraded for inconsistency (I²>50%) or imprecision (CI crossing null or <3 studies) GRADE assessment showing certainty of evidence for coffee consumption and heart failure outcomes across different subgroups. Evidence ranges from very low to moderate certainty; moderate coffee (2-4 cups/day) shows HR 0.925 (95% CI 0.882-0.971, I²=0%) with low certainty based on J shaped dose-response relationship across 7 studies and 656,666 participants.

**Supplementary Table S6.** Non-linear dose-response pooled estimates (restricted cubic spline model)

| Coffee (cups/day) | Pooled HR | 95% CI | No. studies contributing |
| --- | --- | --- | --- |
| 0 | 1.000 | 1.000-1.000 |  |
| 1 | 0.881 | 0.843-0.921 | 4 |
| 1.5 | 0.879 | 0.842-0.918 | 6 |
| 2 | 0.881 | 0.844-0.919 | 6 |
| 2.5 | 0.989 | 0.889-1.102 | 4 |
| 3 | 0.920 | 0.876-0.965 | 7 |
| 3.5 | 0.919 | 0.874-0.966 | 6 |
| 4 | 0.910 | 0.864-0.958 | 5 |
| 4.5 | 0.912 | 0.753-1.104 | 2 |
| 5 | 0.876 | 0.699-1.099 | 3 |
| 5.5 | 0.922 | 0.810-1.050 | 6 |
| 6 | 0.922 | 0.810-1.050 | 6 |
| 8 | 0.886 | 0.742-1.058 | 2 |
| 11 | 0.963 | 0.797-1.163 | 2 |

Dose-response relationship showing pooled hazard ratios with 95% confidence intervals for coffee consumption from 0-11 cups/day using restricted cubic spline modeling. Protective effect observed from 1-8 cups/day with nadir at 2-4 cups (HR ~0.920-0.910), with 2-7 studies contributing to each consumption level.

**Supplementary Table S7.** Sensitivity and reporting checks

| Sensitivity / reporting check | Result |
| --- | --- |
| Studies that only used Never/non-drinkers as the Reference group | Pooled HR was 0.93 (95% CI, 0.80–1.07; P=0.31; I²=45.8%). |
| Leave-one-out (moderate coffee) | Pooled HR range: 0.913 to 0.933 (all statistically significant) |
| Excluding Han et al. (UK Biobank) | HR 0.933 (95% CI 0.841-1.036); I2 = 24.2% |
| Publication bias (Egger test) | z = 0.013; P = 0.99 |

Robustness checks including Studies that only used Never/non-drinkers as the Reference group (Pooled HR was 0.93 (95% CI, 0.80–1.07; P=0.31; I²=45.8%)). leave-one-out analysis for moderate coffee (pooled HR 0.943, 95% CI 0.935-0.935), exclusion of UK Biobank studies (HR 0.933, 95% CI 0.841-1.036, I²=24.2%), and Egger test for publication bias (t=0.015, P=0.99 indicating no significant bias).

**Supplementary Table S8.** UK Biobank Overlap Documentation

| Study | UK Biobank Population | N | Analysis Used |
| --- | --- | --- | --- |
| Han 2023 | General population | 497,503 | Primary analysis |
| Ke 2025 | General population (sex-stratified) | 468,629 | Excluded (overlap) |
| Chieng 2022 | General population (coffee subtypes) | 449,563 | Descriptive only |
| Liu 2023 | T2DM never-smokers | 10,793 | Descriptive only |
| Ma 2025 | T2DM population | 14,277 | Descriptive only |

**Supplementary Table S9.** Reference Group Definitions by Study

| Study | Reference Category | Dose |
| --- | --- | --- |
| Wilhelmsen 2001 | Non-drinkers | 0 |
| Ahmed 2009 | <1 cup/day | 0.5 |
| Levitan 2011 | <1 cup/day | 0.5 |
| Wang 2011 | Non-drinkers | 0 |
| Han 2023 | <1 cup/day | 0.5 |
| Bodar 2020 | Non-drinkers | 0 |


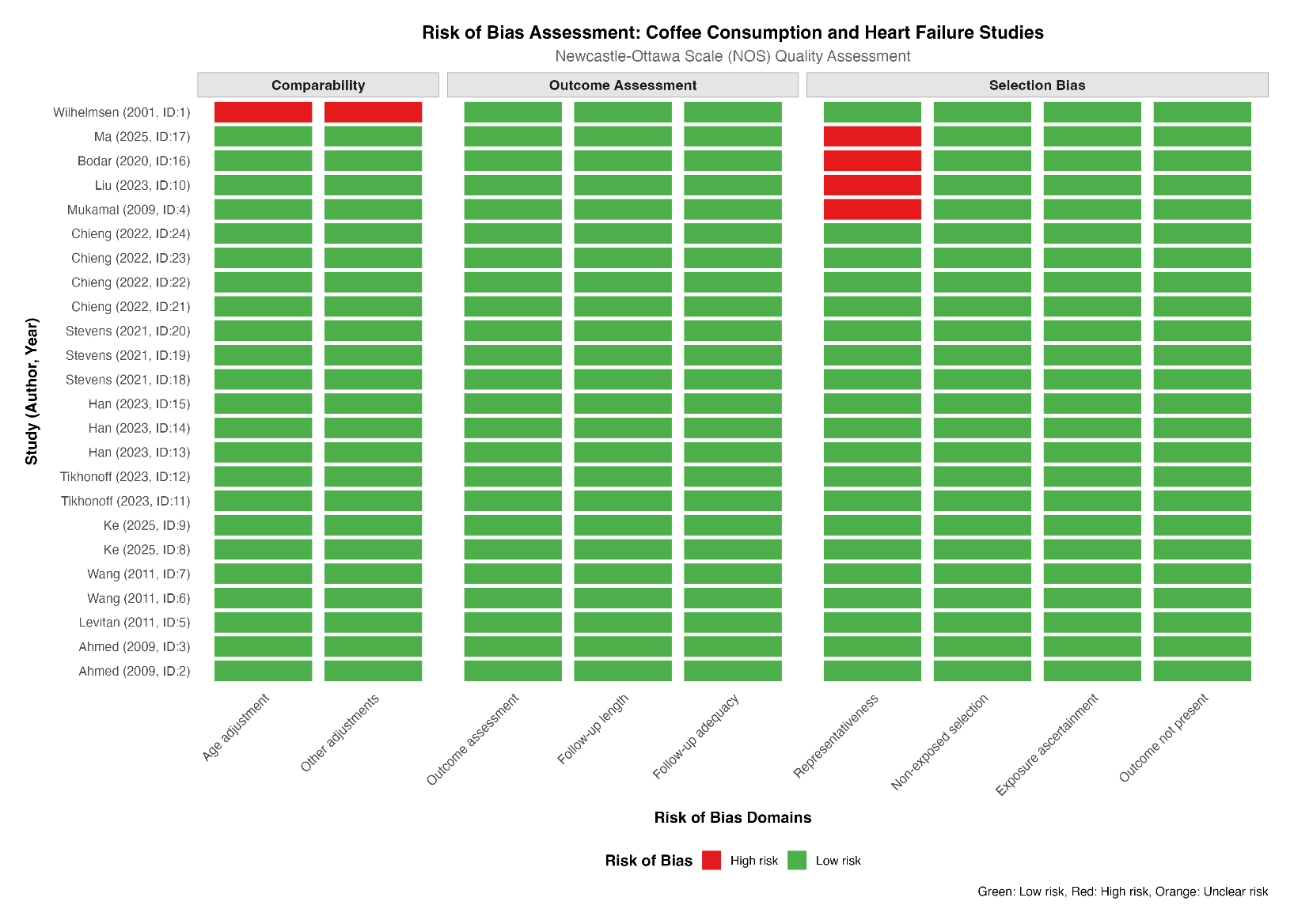
**B\ Supplementary Figures and Figures’ Legends**

**Supplementary Figure S1. Risk of bias traffic-light plot (NOS domains)**: Newcastle-Ottawa Scale quality assessment for included observational studies showing risk of bias across three domains: comparability, outcome assessment, and selection bias. Color-coded visualization: green (low risk), yellow (moderate), red (high risk); most studies demonstrate low-to-moderate overall risk.


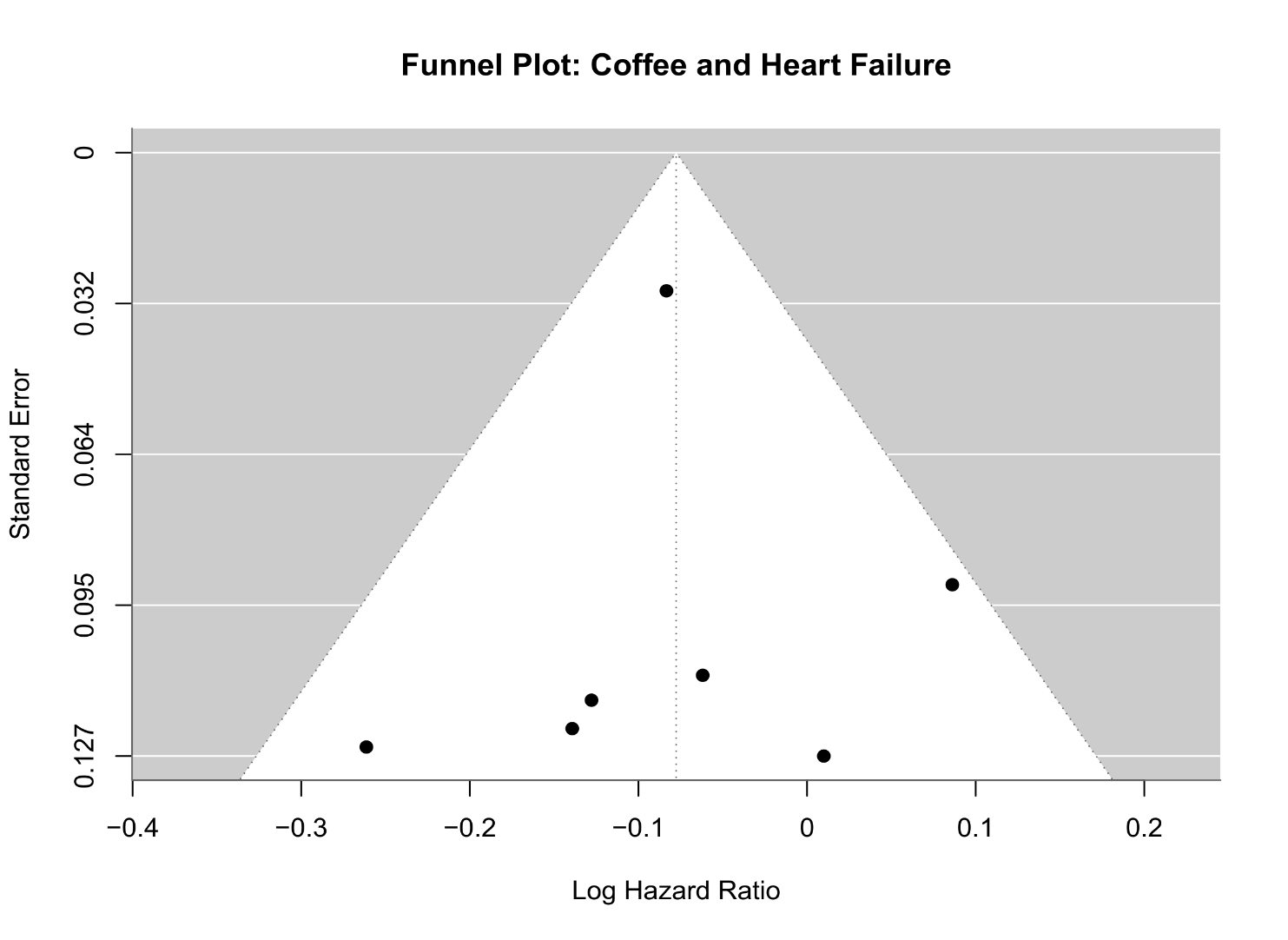
**Supplementary Figure S2. Funnel Plot for Publication Bias Assessment:** Comparison-adjusted funnel plot examining small-study effects and potential publication bias in the coffee-heart failure meta-analysis. Studies distributed symmetrically around pooled estimate (log hazard ratio on x-axis, standard error on y-axis), suggesting minimal publication bias; Egger test non-significant (P=0.99).


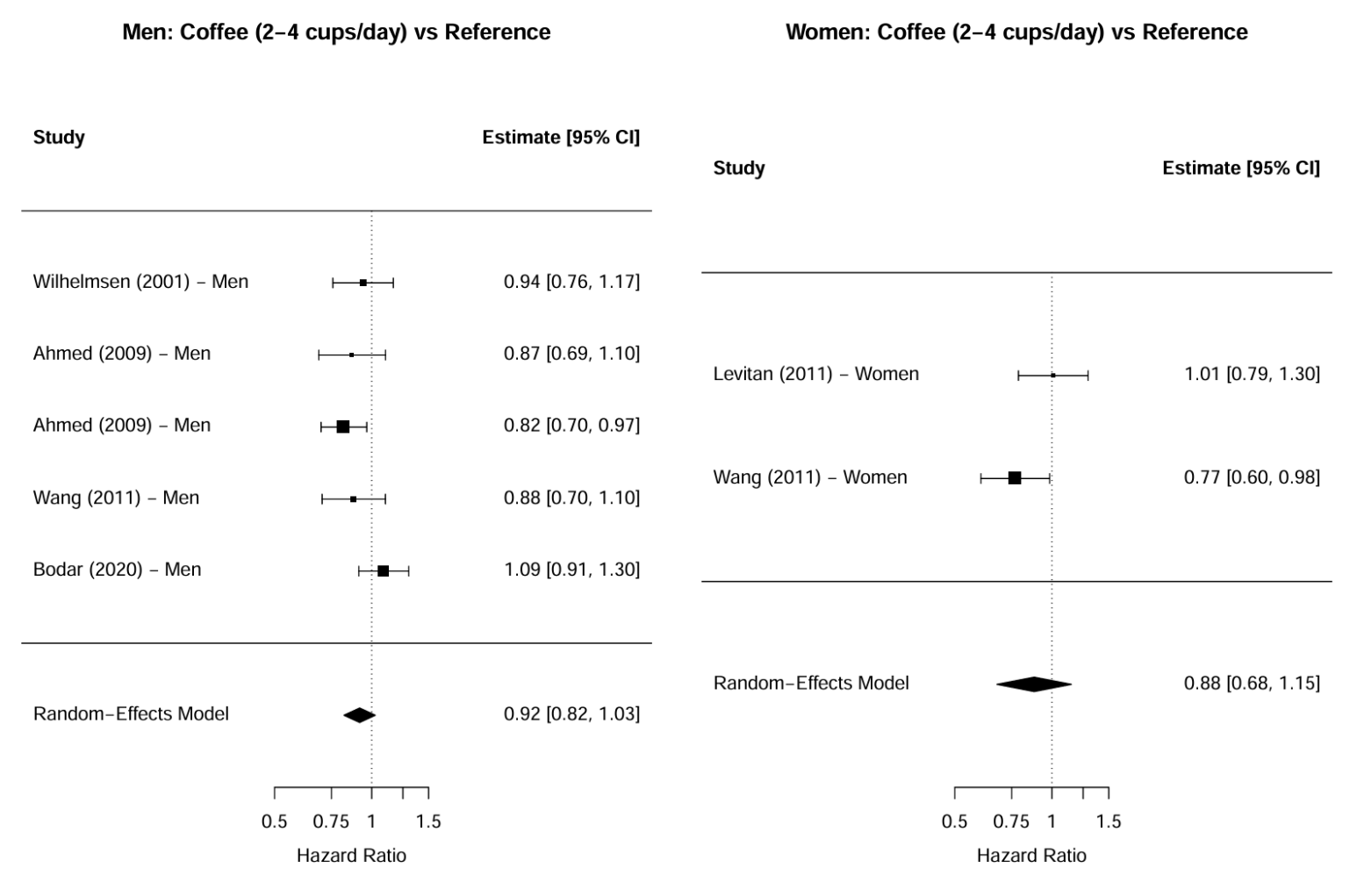
 **Supplementary Figure S3. Sex-stratified Forest plot**: Separate Forest plots for men and women showing study-specific and pooled hazard ratios for moderate coffee consumption (2-4 cups/day) versus reference. Random-effects models show consistent protective associations in both sexes: men HR 0.92 (95% CI 0.82-1.03, P=0.13), women HR 0.88 (95% CI 0.68-1.15, P=0.35). P value for interaction between sexes subgroup (men vs women) was 0.783 so it wasn’t significant.
